# Factors associated with uncontrolled blood pressure among Ghanaians: Evidence from a multicenter hospital-based study

**DOI:** 10.1371/journal.pone.0193494

**Published:** 2018-03-19

**Authors:** Fred Stephen Sarfo, Linda M. Mobula, Gilbert Burnham, Daniel Ansong, Jacob Plange-Rhule, Osei Sarfo-Kantanka, David Ofori-Adjei

**Affiliations:** 1 Department of Medicine, Komfo Anokye Teaching Hospital, Kumasi, Ghana; 2 Department of Medicine, Kwame Nkrumah University of Science and Technology, Kumasi, Ghana; 3 Johns Hopkins University School of Medicine, Baltimore, Maryland, United States of America; 4 Johns Hopkins University Bloomberg School of Public Health, Baltimore, Maryland, United States of America; 5 Ghana College of Physicians and Surgeons, Accra, Ghana; 6 Department of Medicine & Therapeutics, University of Ghana School of Medicine and Dentistry, Accra, Ghana; East Tennessee State University, UNITED STATES

## Abstract

**Background:**

The burden of uncontrolled hypertension in Low-and-Middle Income Countries (LMICs) is high, with an increased risk of cardiovascular diseases and chronic renal failure in these settings.

**Objective:**

To assess the factors associated with uncontrolled blood pressure control in a cross-section of Ghanaian hypertensive subjects involved in an on-going multicenter epidemiological study aimed at improving access to hypertension treatment.

**Methods:**

A cross-sectional study involving 2,870 participants with hypertension with or without diabetes who were enrolled at 5 hospitals in Ghana (2 tertiary, 2 district and 1 rural hospital). Data on demographics, medical history, lifestyle factors, anti-hypertensive medications and treatment adherence were collected. The 14-item version of the Hill-Bone compliance to high blood pressure therapy scale was used to assess adherence to treatment in 3 domains namely adherence to medications, salt intake and clinic appointments. Questionnaires on knowledge, attitudes and practices on hypertension, sources of antihypertensive medications and challenges with accessing these medications were also administered. Blood pressure, weight and height were measured for each subject at enrollment. Factors associated with uncontrolled blood pressure (>140/90mmHg) were assessed using a multivariate logistic regression model.

**Results:**

The mean ± SD age of study participants was 58.9 ± 16.6 years, with a female preponderance (76.8%). Among study participants, 1,213 (42.3%) study participants had blood pressure measurements under control. Factors that remained significantly associated with uncontrolled blood pressure with adjusted OR (95% CI) included receiving therapy at a tertiary level of care: 2.47 (1.57–3.87), longer duration of hypertension diagnosis: 1.01 (1.00–1.03), poor adherence to therapy: 1.21 (1.09–1.35) for each 5 points higher score on the Hill-Bone scale, reported difficulties in obtaining antihypertensive medications: 1.24 (1.02–1.49) and number of antihypertensive medications prescribed: 1.32 (1.21–1.44).

**Conclusion:**

We have found high rates of uncontrolled blood pressure among Ghanaian patients with hypertension accessing healthcare in public institutions. The system-level and individual-level factors associated with poor blood pressure control should be addressed to improve hypertension management among Ghanaians.

## Introduction

Hypertension is a major global public health challenge with nearly 1 billion individuals affected worldwide and projected to reach 1.56 billion by 2025 [1]. Hypertension remains the premiere vascular risk factor for chronic kidney disease and cardiovascular diseases including stroke, myocardial infarction and heart failure which are among the leading causes of global morbidity and mortality [2,3]. Recent estimates have depicted a clear decline in prevalence rates of uncontrolled hypertension in high-income countries over the past 4 decades [4]. In contrast, there has been a significant rise in the burden of uncontrolled hypertension in Low-and-Middle Income Countries (LMICs) in sub-Saharan Africa, south Asia, and central and eastern Europe [4]. The benefits of blood pressure lowering treatment for prevention of cardiovascular disease are well established [5–7]. A meta-analysis has shown that a 10 mm Hg reduction in systolic blood pressure reduced the risk of major cardiovascular disease events by 20%, coronary heart disease by 17%, stroke by 27%, heart failure by 28%, and all-cause mortality by 13% [8].

In LMICs, health systems are not well equipped to provide care for non-communicable diseases (NCDs) such as hypertension which are often life-long diseases requiring sustained access to healthcare for control. Among populations in LMICs, awareness about hypertension control and the need for continuous treatment are low. Many other factors including limited resource allocation, lack of training in hypertension treatment among many professionals, and low health literacy among patients contribute to poor blood pressure control. These factors have contrived to create a looming epidemic of Cardiovascular Diseases (CVDs) in these settings. One of the key Sustainable Development Goals adopted by the World Health Assembly in 2013 was to lower the prevalence of raised blood pressure by 25% by 2025 [9]. In order to meet this target, data on the underpinning factors associated with blood pressure control particularly from LMICs are needed to understand the contextual and systemic factors promoting the burgeoning CVD epidemic in these regions.

To address the aforementioned challenges in blood pressure control in LMICs, both community and hospital based studies are urgently needed. Such studies will provide the needed information on the key local drivers of the poor rates of hypertension control [10–13] and help formulate strategic interventions to improve blood pressure control. An understanding of the contributions of individual level and health service delivery factors for the control of hypertension would be vital for designing and testing population level interventions for the management of hypertension and also serve as a model for management of other NCDs in SSA. Conceptually, patient-level factors such as demographic and socio-economic characteristics, lifestyle and behavioral indicators, health literacy and adherence to hypertension treatment may contribute to differential extents to poor blood pressure control. Additionally, system-level factors such as availability and affordability of antihypertensive medications and the level of competence of healthcare practitioners on hypertension management together with prevailing cultural beliefs and practices could all influence blood pressure control in SSA.

The Ghana Access and Affordability Program (GAAP), a public-private partnership aims to improve the management of hypertension and type II diabetes through improved access to medicines for the control of these conditions. In this report we present a comprehensive analysis of factors associated with blood pressure control among 2,870 Ghanaian hypertensive patients enrolled as part of the GAAP pilot study. Our objective was to understand the unique factors associated with uncontrolled blood pressure among Ghanaians with hypertension under routine health delivery settings. The patient population in the present study received medical care from clinics located in primary, secondary and tertiary facilities across the country.

## Methods

### Ethical permission

This study protocol was approved by the Committee on Human Research Publications and Ethics of the Kwame Nkrumah University of Science and Technology and the Ghana Health Services Ethical Review Committee. It was declared as exempt by the Institutional Review Board at the Johns Hopkins Bloomberg School of Medicine. Written informed consent was obtained from all study participants before enrollment into the study. All relevant data are included in the manuscript and S1 Table.

### Study design

The present report is a cross-sectional analysis of baseline data of a prospective cohort study of hypertensive and diabetic patients in the GAAP pilot study.

### Study sites

The GAAP Pilot study was conducted at 5 hypertension and diabetes specialty and general clinics in urban, peri-urban and rural locations in Ghana. These sites were conveniently selected based on the ecological zones of northern savannah, central forest -mixed zone and the coastal belt. Briefly, the study sites included:

- The Agogo Presbyterian Hospital, (APH) is a peri-urban, district, secondary level health institution situated in the middle belt of Ghana.
- The Atua Government Hospital, (AGH) is a peri-urban, district, secondary level health institution situated in the southern belt of Ghana.
- The Komfo Anokye Teaching Hospital, (KATH) is an urban, tertiary level health institution situated in the middle belt of Ghana,
- The Kings Medical center, (KMC) is a rural, primary level health institution situated in the Northern belt of Ghana.
- The Tamale Teaching Hospital, (TTH) is an urban, tertiary level health institution situated in the Northern belt of Ghana.

### Recruitment of study participants

Participants were eligible if they were 18 years or older with known diagnosis of hypertension and/or type II diabetes presenting for routine care at either a general polyclinic (AGH, KMC, TTH) or a dedicated diabetes/hypertension clinic, (KATH, APH). Participants were excluded if they had hypertensive urgency/emergency or glycemic complications such as hypoglycemia or a hyperglycemic emergency at initial contact for enrollment. During the 6-month enrollment period of the study, each consecutive participant meeting the eligibility criteria was invited for enrollment by research assistants after explaining the objectives of study and obtaining informed consent.

### Evaluation of study participants

Trained Research Assistants interviewed study participants and collected demographic and household information such as age, gender, educational attainment, employment status, number of dependents on monthly income and health expenditures. Study participants were also interviewed on their lifestyle behaviors such as alcohol use, cigarette smoking, and level of physical activities. A detailed medical history including duration of hypertension or diabetes diagnosis and compliance with hypertension treatment was assessed using the 14-item version of Hill-Bone compliance to high blood pressure therapy scale which has demonstrated good validity and internal reliability [14–17]. The scale has 3 subscales under behavioral domains of hypertension treatment including medication adherence, reduced salt intake and appointment keeping with each question/item answered with a four-point Likert scale ranging from 1 to 4 (1 = none of time, 2 = some of the time, 3 = most of time, and 4 = all the time) [14]. Total score ranged from 14 (perfect adherence) to 56 (non-adherence) with higher scores denoting overall poorer adherence. We also assessed and documented whether there were difficulties in obtaining blood pressure medications for disease control through structured interviews using questionnaires. Furthermore the knowledge, attitudes and practices on hypertension of participants were assessed using seven close-ended questions.

The vital signs of study participants including measurements of blood pressure and pulse rates were performed by trained study nurses following a standardized study protocol. Briefly, each site was provided with an automated blood pressure measurement device (Omron HEM-907XL) for blood pressure and pulse measurements. Each study participant rested for at least 5 minutes prior to blood pressure measurements while sitting in a chair with both feet flat on the floor. Both arms were supported at the level of the heart on a table. Two consecutive blood pressure readings from the same arm taken 2 minutes apart were recorded and averaged for the present analysis.

Anthropometric evaluations including measurement of weight, height and waist circumference were performed by Study nurses. Weight was measured using a pre-calibrated Secca scale with participants wearing light clothing and barefooted. Weight was rounded off to the nearest 1 kg. Height was measured with the participant standing upright against a wall using a previously affixed height measuring device. Body mass index (BMI) of each participant was then derived by dividing the weight in kilograms by the square of the height in meters. Waist circumference was measured in the mid-axillary line midway between the lower rib and the superior iliac crest using a standard non-stretchable tape.

Alcohol intake was categorized into never, former drinker or current drinker. Quantity of alcohol consumed by current drinkers was classified according to amount of alcohol ingested in the past month into daily, 5–6 days per week, 3–4 days per week, 1–2 days per week and 1–3 days per month. Cigarette smoking status was defined as never, former, or current smoker. We defined current smokers as individuals who smoked any tobacco in the past 12 months and included those who had quit within the past year. Former smokers were defined as those who had quit more than a year earlier. Physical activity was assessed by asking if participants frequently performed physical activities that caused a small increase in breathing or made their heart rate increase, such as (fast/brisk) walking, jogging, bicycling and how much they spent during physical activity.

Stroke was self-reported if participant had ever experienced sudden onset of weakness or sensory loss on one side of the body, sudden loss of vision, or sudden loss of speech. Heart failure was self reported if participant had ever experienced shortness of breath on exertion, on lying down as well as swelling of both feet.

### Laboratory measurements

To ensure standardization across all study sites, an International Organization for Standardization (ISO)-certified and quality-assured laboratory was contracted to run all biochemical panels which included serum creatinine, lipid profile and hemoglobin A1C for subjects with diabetes. Samples were transported to the laboratory by trained phlebotomists on the same day of collection often within 4 hours or where not feasible (KMC and AGH sites), samples were stored in a freezer before transported to the laboratory the next day.

### Statistical analysis

Means and medians were compared using either the Student’s t-test or Mann-Whitney’s U-test for paired comparisons. Proportions were compared using the Chi-squared tests or Fisher’s exact test for proportions with subgroupings <5. Correlations between two continuous variables were assessed using the Pearson’s correlation coefficient. A multivariate logistic regression analysis was performed to identify factors independently associated with the risk of clinic blood pressure reading not on target of <140/90mmHg. We evaluated blood pressure control as a binary variable—poor control or well controlled. Independent variables evaluated included the following categorical variables: age (categorized as <60 years vs ≥60 years), gender, monthly income, location of residence (categorized as urban, peri-urban and rural), level of healthcare institution (primary, district/secondary and tertiary levels), comorbid diabetes status and employment status. The following continuous variables were evaluated in the model: duration of hypertension history, number of antihypertensives prescribed, scores on 14-item version of Hill-Bone compliance to high blood pressure therapy scale. In building our model, factors associated with the outcome variable at a p-value of <0.05 in unadjusted analyses were included in the multivariate analysis. We investigated putative factors associated with poor blood pressure control among Ghanaians based on literature search, clinical understanding of hypertension management and empirical evidence from our data (significant associations observed in bivariate analysis). In all analyses, two-tailed p-values <0.05 were considered statistically significant. Statistical analysis was performed using SPSS version 19.

## Results

### Socio-demographic characteristics of study participants

The present analysis involves 2,870 participants, of which 1685 (65.0%) were known to have hypertension alone and 1,005 (35.0%) had a dual diagnosis of type II diabetes and hypertension. The mean (± SD) age of study participants was 58.9 ± 16.6 years, with a female preponderance (76.8%). It was found that 41.7% resided in urban locations, 22.0% in semi-urban and 36.2% in rural dwellings. As shown in Table 1, approximately 55% had no formal education or primary level education, 34% had attained secondary education and 11% had tertiary education. Nearly 54% of study participants were recruited from the two tertiary referral centers (KATH and TTH), 40% were recruited from the two district-level hospitals (APH and AGH) and the remaining 6% were recruited from a primary level rural hospital. 2851 (99.3%) study participants had valid National Health Insurance Scheme (NHIS) cards with access to generic brands of antihypertensive medications with rates of 99.6% at AGH (n = 280), 99.3% at APH (n = 873), 99.7% at KATH (n = 1,156), 98.3% at KMC (n = 174) and 98.7% at TTH (n = 387).

**Table 1:** Comparison of characteristics of study participants according to blood pressure control status.

Characteristic	Blood pressure <140/90mmHg N = 1,213 (42.3%)	Blood pressure >140/90mmHg N = 1,657 (57.7%)	Total N = 2,870	p-values
**Age, mean ± SD**	58.9 ± 20.9	58.9 ± 12.7	58.9 ± 16.6	0.93
**Female, n (%)**	932 (76.8)	1273 (76.8)	2205 (76.8)	1.00
**Location of residence**				0.0003
Urban	470 (38.7)	728 (43.9)	1198 (41.7)	
Semi-urban	252 (20.8)	380 (22.9)	632 (22.0)	
Rural	491 (40.5)	549 (33.1)	1040 (36.2)	
**Highest Educational status**				0.50
No formal education	355 (29.3)	509 (30.7)	864 (30.1)	
Primary level	297 (24.5)	408 (24.6)	705 (24.6)	
Secondary level	407 (33.6)	565 (34.1)	972 (33.9)	
Tertiary level or more	153 (12.6)	174 (10.5)	327 (11.4)	
No response	1 (0.1)	1 (0.1)	2 (0.1)	
**Employment status**				0.03
Unemployed	278 (22.9)	443 (26.7)	721 (25.1)	
Retired	100 (8.2)	134 (8.1)	234 (8.2)	
Self-employed	296 (24.4)	373 (22.5)	669 (23.3)	
Farming	179 (14.8)	201 (12.1)	380 (13.2)	
Trading	198 (16.3)	295 (17.8)	493 (17.2)	
Government employee	104 (8.6)	113 (6.8)	217 (7.6)	
Others	58 (4.8)	98 (5.9)	156 (5.4)	
**Monthly Household income**				0.94
>1,000 GHc	86 (7.1)	128 (7.7)	214 (7.5)	
500–1,000 GHc	108 (8.9)	153 (9.2)	261 (9.1)	
300–500 GHc	139 (11.5)	174 (10.5)	313 (10.9)	
210–300 GHc	77 (6.3)	100 (6.0)	177 (6.2)	
<210 GHc	466 (38.4)	635 (38.3)	1101 (38.4)	
No response/unknown	337 (27.8)	467 (28.2)	804 (28.0)	
**Level of Health Institution**				<0.0001
Tertiary level	588 (48.5)	955 (57.6)	1543 (53.8)	
Secondary level	527 (43.4)	626 (37.8)	1153 (40.2)	
Primary level	98 (8.1)	76 (4.6)	174 (6.1)	
**Known Hypertensive only, n (%)**	810 (66.8)	1055 (63.7)	1865 (65.0)	0.08
**Known Hypertensive & Diabetic, n (%)**	403 (33.2)	602 (36.3)	1005 (35.0)	
Duration of hypertension, (years)	7.4 ± 6.9	8.5 ± 7.7	8.1 ± 7.4	0.0002
Duration of diabetes mellitus, (years)	9.1 ± 6.0	10.4 ± 7.9	9.9 ± 7.2	0.007
**Lifestyle/Behavioral factors**				
Current alcohol use	95 (7.8)	124 (7.4)	219 (7.6)	0.73
Current cigarette smoking	8 (0.7)	6 (0.4)	14 (0.5)	0.26
Previous cigarette use	91 (7.5)	104 (6.3)	195 (6.8)	0.20
Regular Physical activity	736 (60.7)	994 (60.0)	1730 (60.3)	0.74
Daily duration of physical activity (minutes), mean ± SD	18.9 ± 23.0	19.0 ± 23.6	19.0 ± 23.3	0.93
Body Mass Index, mean ± SD	26.7 ± 5.6	26.7 ± 5.4	26.7 ± 5.5	0.85
Waist Circumference, mean ± SD	96.0 ± 13.0	96.1 ± 12.6	96.1 ± 12.8	0.74
**Laboratory Data**				
Serum creatinine, mean ± SD	78.9 ± 38.9	86.1 ± 64.6	83.0 ± 55.3	0.001
eGFR, mean ± SD	77.5 ± 15.0	74.6 ± 17.6	75.8 ± 16.6	<0.0001
HBA1C, mean ± SD	8.4 ± 2.5	8.4 ± 2.4	8.4 ± 2.4	0.96
Serum total cholesterol, mean ± SD	5.2 ± 1.3	5.5 ± 1.3	5.4 ± 1.3	0.01
LDL cholesterol, mean ± SD	3.28 ± 1.11	3.53 ± 1.20	3.43 ± 1.18	0.02
HDL cholesterol, mean ± SD	1.33 ± 0.57	1.34 ± 0.52	1.33 ± 0.54	0.83
Triglyceride, mean ± SD	1.55 ± 0.83	1.63 ± 1.10	1.60 ± 1.00	0.40
**Hill-Bone Adherence scores**				
Total scores	20.4 ± 3.4	20.9 ± 4.0	20.7 ± 3.8	0.002
Medication adherence	8.5 ± 2.1	8.8 ± 2.5	8.7 ± 2.4	0.0009
Salt usage	4.6 ± 1.6	4.5 ± 1.5	4.5 ± 1.6	0.74
Appointment keeping	7.4 ± 1.2	7.6 ± 1.4	7.5 ± 1.4	0.0006

eGFR = estimated Glomerular filtration rate assessed using the CKD-EPI formula.

### Demographic and clinical disposition according to blood pressure control targets

1,213 (42.3%) study participants had blood pressure measurements below 140/90mm Hg and were considered to be on target whilst the remainder (57.7%) were classified as uncontrolled. There were no significant differences between the two groups with respect to age, gender, educational status or household monthly income as shown in Table 1. However, participants whose blood pressure were not on target were more likely to reside in urban dwellings, to receive care in a tertiary health institution, less likely to be employed, and had a longer duration of diagnosis of hypertension and co-morbid diabetes mellitus. Current alcohol intake and cigarette use were reported at 7.6% and 0.5% respectively among the entire cohort with no significant differences between those whose blood pressure were on target and those who were not. Self reported rates of stroke and heart failure were 5.4% and 6.0% respectively with no differences between the two groups. The mean estimated glomerular filtration rate (eGFR) was 74.6 ± 17.6 ml/min among subjects not on blood pressure control target versus 77.5% ± 15.0 ml/min among those on target, p<0.0001. Furthermore total serum cholesterol and LDL-cholesterol were significantly higher among those with uncontrolled blood pressure, although serum HDL-cholesterol and triglyceride concentrations were comparable between the two groups.

### Adherence to high blood pressure therapy

Using the 14-item version of the Hill-Bone Adherence scale we found that most of study participants were moderately adherent to therapy with a mean score of 20.7 ± 3.8. Adherence scores were significantly higher among those with uncontrolled hypertension (20.9±4.0) compared with a score 20.4 ± 3.4 among those on target, p = 0.002. Overall, average scores on adherence to medications and appointment keeping domains of the Hill-Bone scale were significantly poorer among the group with poorly controlled hypertension than those with controlled blood pressure as shown in Table 2.

**Table 2:** Responses to the Hill-Bone compliance to high blood pressure therapy scale.

ITEMS	Blood pressure <140/90mmHg				Blood pressure >140/90mmHg				P-value
	None of the time	Some of the time	Most of the time	All of the time	None of the time	Some of the time	Most of the time	All of the time	
1. Patients forget to take their hypertension medicine	878 (72.4)	239 (19.7)	21 (1.7)	24 (2.0)	1201 (72.5)	314 (18.9)	52 (3.1)	45 (2.7)	0.07
2. Patients decide not to take their hypertension medicine	940 (77.5)	180 (14.8)	24 (2.0)	18 (1.4)	1257 (75.9)	265 (16.0)	41 (2.5)	50 (3.0)	0.04
3. Patients eat salty food	569 (46.9)	354 (29.2)	98 (8.1)	143 (11.8)	825 (49.8)	458 (27.6)	141 (8.5)	193 (11.6)	0.62
4. Patients add salt to their food before they ate it	973 (80.2)	132 (10.9)	37 (3.1)	22 (1.8)	1348 (81.4)	191 (11.5)	57 (3.4)	22 (1.3)	0.67
5. Patients eat fast food (food purchased for take-out)	687 (56.6)	408 (33.6)	54 (4.5)	15 (1.2)	964 (58.2)	532 (32.1)	92 (5.5)	27 (1.7)	0.39
6. Clinic make scheduled return appointments before patients left the doctor’s office	49 (4.0)	37 (3.5)	50 (4.1)	1026 (84.6)	70 (4.2)	60 (3.6)	50 (3.1)	1432 (86.4)	0.35
7. Patients miss scheduled clinic appointments	936 (77.2)	179 (14.8)	20 (1.6)	26 (2.1)	1227 (74.0)	299 (18.0)	48 (2.9)	39 (2.4)	0.02
8. Patients forget to get prescriptions filled	1040 (85.7)	93 (7.7)	9 (0.7)	20 (1.6)	1380 (83.3)	170 (10.3)	17 (1.0)	46 (2.8)	0.02
9. Patients run out of High Blood Pressure pills	945 (77.9)	195 (16.1)	14 (1.2)	8 (0.7)	1252 (75.6)	305 (18.4)	38 (2.3)	18 (1.1)	0.03
10. Patients skip their High Blood Pressure medicine before going to the doctor	781 (64.4)	175 (14.4)	27 (2.2)	179 (14.8)	1009 (60.9)	280 (16.9)	66 (4.0)	258 (15.6)	0.01
11. Patients miss taking their High Blood Pressure pills when they felt better	1079 (89.0)	70 (5.7)	8 (0.7)	5 (0.4)	1451 (87.6)	119 (7.2)	28 (1.7)	15 (0.9)	0.01
12. Patients missed taking their High Blood Pressure pills when they felt sick	1076 (88.7)	62 (5.1)	12 (1.0)	4 (0.3)	1469 (88.7)	84 (5.1)	30 (1.8)	12 (0.7)	0.16
13. Patients took someone else’s High Blood Pressure pills	1134 (93.5)	27 (2.2)	3 (0.2)	0 (0.0)	1569 (94.6)	39 (2.4)	2 (0.1)	1 (0.0)	0.70
14. Patients missed taking their high Blood Pressure pills when there were careless	1074 (88.5)	81 (6.7)	4 (0.3)	2 (0.2)	1437 (86.7)	141 (8.5)	24 (1.4)	10 (0.6)	0.002

### Knowledge, attitudes and practices on hypertension

Among study participants, 63.2% knew the meaning of the term hypertension but only 6.2% and 3.1% respectively had accurate knowledge on what the systolic and diastolic blood pressure cut-offs should be for hypertension diagnosis. As shown in Table 3, nearly 87% thought hypertension was a very serious health concern and 86% thought that it was very important to take medications to keep blood pressure under control. Also, 47.4% thought hypertension could aggravate risk of stroke, heart attack (13.7%) and kidney diseases (2.3%) respectively. Interestingly, we found significantly better responses among participants whose blood pressure were not on target regarding their knowledge on the factors most important in controlling blood pressure such as exercising, reducing stress, reducing salt intake and losing weight compared with those on target. (Table 3).

**Table 3:** Knowledge, attitudes & practices of patients on hypertension according to BP control status.

QUESTION	Blood pressure <140/90mmHg N = 1,213	Blood pressure >140/90mmHg N = 1,657	Total N = 2870	P-values
1. What does the term hypertension mean?				0.60
Raised blood pressure	773 (63.7)	1040 (62.8)	1813 (63.2)	
2. What should be the upper/systolic blood pressure reading be to a hypertensive?				
>140mmHg	70 (5.8)	108 (6.5)	178 (6.2)	0.70
= 140mmHg	26 (2.1)	42 (2.5)	68 (2.4)	
<140mmHg	415 (34.2)	546 (33.0)	961 (33.5)	
I don’t know	702 (57.9)	961 (58.0)	1663 (57.9)	
3. What should be the lower/diastolic blood pressure reading be to a hypertensive?				<0.0001
>90mmHg	68 (5.6)	21 (1.3)	89 (3.1)	
= 90mmHg	61 (5.0)	55 (3.3)	116 (4.0)	
<90mmHg	88 (7.3)	856 (51.7)	944 (32.9)	
I don’t know	996 (82.1)	725 (43.8)	1721 (60.0)	
4. How serious of a health concern has high blood pressure been to you?				<0.0001
Not at all serious	19 (1.6)	65 (3.9)	84 (2.9)	
Somewhat serious concern	81 (6.7)	136 (8.2)	217 (7.6)	
Very serious concern	1034 (85.2)	1456 (87.9)	2490 (86.8)	
No response	79 (6.5)	0 (0.0)	79 (2.8)	
5. How important do you think taking medicine is to keeping blood pressure under control?				<0.0001
Not at all important	12 (1.0)	19 (1.1)	31 (1.1)	
Somewhat important	95 (7.8)	188 (11.3)	283 (9.7)	
Very important	1025 (84.5)	1447 (87.3)	2472 (86.1)	
No response	81 (6.7)	3 (0.2)	84 (2.9)	
6. What are the most important factors in controlling your blood pressure?				
Taking medications	698 (57.5)	903 (54.5)	1601 (55.8)	0.10
exercising	357 (29.4)	684 (41.3)	1041 (36.3)	<0.0001
Less stress	194 (16.0)	403 (24.3)	597 (20.8)	<0.0001
Quitting smoking if you are smoking	61 (5.0)	88 (5.3)	149 (5.2)	0.74
Reducing salt intake in your diet	502 (41.4)	784 (47.3)	1286 (44.8)	<0.0001
Reducing alcohol intake	76 (6.3)	139 (8.4)	215 (7.5)	0.03
Losing weight	52 (4.2)	120 (7.2)	172 (6.0)	0.001
All of the above	61 (5.0)	63 (3.8)	124 (4.3)	0.11
I don’t know	204 (16.8)	367 (22.1)	571 (19.9)	0.0004
7. High blood pressure can worsen or increase				
The risk of a heart attack	155 (12.8)	239 (14.4)	394 (13.7)	0.21
The risk of stroke	479 (39.5)	882 (53.2)	1361 (47.4)	<0.0001
The risk of kidney problems	25 (2.1)	42 (2.5)	67 (2.3)	0.41
All the above	66 (5.4)	101 (6.1)	167 (5.8)	0.46
I don’t know	247 (20.4)	433 (26.1)	680 (23.7)	0.0003

### Barriers to antihypertensive medication access

98% of study participants obtained their antihypertensive medications from hospital pharmacy at the study sites. In addition, 35% and 7% obtained their medications from Community Pharmacies and Licensed Chemical sellers respectively when medications were not available in the hospital pharmacy. Nearly 52% of participants reported being referred from the Hospital pharmacy to either a Community Pharmacy or Licensed Chemical seller for their antihypertensive medications at some point over the preceding 6 months. Over the previous 6 months, 20.5% reported experiencing difficulty obtaining medications at hospital pharmacies, 6.5% at community pharmacy and 1% at Licensed chemical seller as shown in Table 4. Commonly encountered difficulties included unavailability of medications at the point of care and medications being too expensive when not covered by insurance. 431 (15%) participants reported ever treating hypertension with alternative forms of treatment such as using herbal medicines, homeopathy, acupuncture, traditional healing, resorting to prayer camps and so forth. Patients whose blood pressure were not on target were more likely compared with those on target to report encountering difficulties obtaining their antihypertensive medications 22.5% vs 16.7%, p<0.0001 and were more likely to referred for medications outside a hospital pharmacy 57.8% vs 44.8%, p<0.0001 respectively.

**Table 4:** Barriers to accessing antihypertensive medications among Ghanaian hypertensive participants.

	Blood pressure <140/90mmHg N = 1,213	Blood pressure >140/90mmHg N = 1,657	Total N = 2870	P-values
1. Source of antihypertensive medications*				<0.0001
Hospital pharmacy	1,199 (98.8)	1,612 (97.3)	2811 (97.9)	
Community Pharmacy	332 (27.4)	675 (40.7)	1007 (35.1)	
Licensed Chemical seller	69 (5.7)	120 (7.2)	189 (6.6)	
Other	0 (0.0)	6 (0.4)	6 (0.2)	
2. Have you had problems obtaining antihypertensive medications?				<0.0001
Yes	202 (16.7)	373 (22.5)	575 (20.0)	
No	929 (76.6)	1216 (73.4)	2145 (74.7)	
No response/NA	82 (6.8)	68 (4.1)	150 (5.2)	
3. Where did you encounter difficulty obtaining your antihypertensive medications in the past 6 months?				0.13
Hospital pharmacy	230 (19.0)	358 (21.6)	588 (20.5)	
Community Pharmacy	65 (5.4)	122 (7.4)	187 (6.5)	
Licensed Chemical seller	13 (1.1)	12 (0.7)	25 (0.9)	
Other	0 (0.0)	4 (0.2)	4 (0.1)	
4. What kind of difficulty did you encounter in obtaining your antihypertensive medication?				0.03
Medications not available at point of care	226 (18.6)	352 (21.2)	578 (20.1)	
Too expensive	21 (1.7)	45 (2.7)	66 (2.3)	
NHIS not accepted at pharmacy	20 (1.6)	27 (1.6)	47 (1.6)	
Others	47 (3.9)	98 (5.9)	145 (5.1)	
5. In the past 6 months, have you ever tried to treat your high blood pressure with an alternative method?	195 (16.1)	236 (14.2)	431 (15.0)	0.17
Herbal medicine	180 (14.8)	231 (13.9)	411 (14.3)	
Homeopathy	2 (0.2)	0 (0.0)	2 (0.1)	
Acupuncture	1 (0.1)	0 (0.0)	1 (0.0)	
Traditional healer	17 (1.4)	5 (0.3)	22 (0.8)	
Prayer camp	13 (1.1)	7 (0.4)	20 (0.7)	
Other	1 (0.1)	3 (0.2)	4 (0.1)	
6. In the past 6 months, have you been referred to a Licensed Chemical seller or Community Pharmacy because your antihypertensive medicine was not available at the hospital pharmacy?				<0.0001
Yes	544 (44.8)	957 (57.8)	1501 (52.3)	
No	669 (55.2)	700 (42.2)	1369 (47.7)	
7. If, yes to question 6, please indicate place of referral to obtain medicines.				<0.0001
Community Pharmacy	394 (32.5)	807 (48.7)	1201 (41.8)	
Licensed chemical seller	164 (13.5)	192 (11.6)	356 (12.4)	
Others	3 (0.2)	2 (0.1)	5 (0.2)	

* An individual could have more than one source for obtaining antihypertensive medications

### Classes of antihypertensive medications used to control blood pressure

In decreasing order, commonly prescribed classes of antihypertensive medications included Calcium channel blockers (82.2%), Angiotensin converting enzyme inhibitors (43.2%), Angiotensin receptor blockers (27.3%), diuretics (26.3%), centrally acting agents (13.9%), beta blockers (9.8%) and peripheral vasodilators (1.5%). The median (IQR) number of antihypertensive medications per participant was 2 (2–3). Notably, participants whose blood pressure was uncontrolled were on a significantly higher number of antihypertensive medications, mean (±SD) of 2.20 ± 0.95 compared with 1.90 ± 0.94, p<0.0001 among those with controlled blood pressure as shown in Table 5. There was no correlation between number of antihypertensive medications subjects were taking and the composite score on the Hill Bone questionnaire, Pearson r = -0.01, p = 0.48.

**Table 5:** Antihypertensive drug classes and combinations according to BP control status.

CLASSES OF ANTIHYPERTENSIVE MEDICATIONS	Blood pressure <140/90mmHg N = 1,213	Blood pressure >140/90mmHg N = 1,657	Total N = 2870	P-values
Angiotensin Converting Enzyme Inhibitors	476 (39.2)	763 (46.0)	1239 (43.2)	0.0003
Angiotensin Receptor Blockers	312 (25.7)	472 (28.5)	784 (27.3)	0.10
Beta blockers	91 (7.5)	189 (11.4)	280 (9.8)	0.0005
Calcium Channel Blockers	961 (79.2)	1398 (84.4)	2359 (82.2)	0.0004
Diuretics	342 (28.2)	498 (30.1)	840 (26.3)	0.28
Centrally acting vasodilators (Methyldopa)	116 (9.6)	282 (17.0)	398 (13.9)	<0.0001
Peripheral vasodilators (Hydrallazine)	7 (0.6)	35 (2.1)	42 (1.5)	0.0007
**NUMBER OF ANTIHYPERTENSIVE MEDICATIONS**				
				<0.0001
1	308 (25.4)	275 (16.6)	583 (20.3)	
2	560 (46.2)	772 (46.6)	1332 (46.4)	
3	220 (18.1)	429 (25.9)	649 (22.6)	
4	43 (3.5)	107 (6.5)	150 (5.2)	
5	9 (0.7)	18 (1.1)	27 (0.9)	
6	0 (0.0)	1 (0.1)	1 (0.0)	
7	0 (0.0)	1 (0.1)	1 (0.0)	
No data available	73 (6.0)	54 (3.3)	127 (4.4)	
Mean ± SD	1.90 ± 0.94	2.20 ± 0.95	2.07 ± 0.95	<0.0001
Median (IQR)	2 (1–2)	2 (2–3)	2 (2–3)	<0.0001

### Factors associated with poor blood pressure control among Ghanaians

Variables significantly associated with poor blood pressure control in unadjusted analyses included location of residence, employment status, level of health care, duration of hypertension diagnosis in years, adherence to hypertension therapy, reported difficulties in obtaining antihypertensive medications and the number of antihypertensive medications subjects were taking as shown in Table 6. The factors that remained significantly associated with poor blood pressure control with adjusted OR (95% CI) were receiving therapy at a tertiary level of care: 2.47 (1.57–3.87), longer duration of hypertension diagnosis: 1.01 (1.00–1.03), poorer adherence scores: 1.21 (1.09–1.35) for each 5 points higher score on the Hill-Bone scale, reported difficulties in obtaining antihypertensive medications: 1.24 (1.02–1.49) and number of antihypertensive medications prescribed: 1.32 (1.21–1.44). Although location of residence was strongly associated with blood pressure control in unadjusted analysis, the effect of location was moderated into non-significance in adjusted analysis possibly due to interactions with level of healthcare where hypertension treatment is received.

**Table 6:** Factors associated with suboptimal blood pressure among Ghanaians.

Associated Factors	Unadjusted OR (95% CI)	P-value	Adjusted OR (95% CI)	P-value
Age				
≥60 years	1.13 (0.97–1.31)	0.11	-	-
<60 years	1.00			
Gender				
Male	0.97 (0.81–1.16)	0.74	-	-
Female	1.00			
Location of residence				
Urban	1.38 (1.17–1.64)	0.0002	0.99 (0.77–1.29)	0.96
Peri-urban	1.35 (1.10–1.65)	0.003	0.89 (0.68–1.16)	0.38
Rural	1.00		1.00	
Employment status				
Not gainfully employed	1.18 (1.01–1.38)	0.04	1.09 (0.92–1.29)	0.30
Gainfully employed	1.00		1.00	
Monthly income level				
>500Ghc	1.05 (0.84–1.30)	0.69	-	-
210–500 Ghc	0.92 (0.74–1.13)	0.42		
<210 Ghc	1.00			
Level of healthcare				
Tertiary referral center	2.09 (1.52–2.87)	<0.0001	2.47 (1.57–3.87)	0.0001
District level	1.53 (1.11–2.11)	0.009	1.27 (0.88–1.83)	0.20
Primary level	1.00		1.00	
Comorbid diabetes mellitus	1.15 (0.98–1.34)	0.08	-	-
Duration of hypertension history	1.18 (1.01–1.03)	0.0002	1.01 (1.00–1.03)	0.02
Adherence score on Hill Bone scale, each 5 points higher	1.18 (1.06–1.31)	0.002	1.21 (1.09–1.35)	0.0006
Reported difficulty in obtaining antihypertensive medications at hospital pharmacy**	1.20 (1.01–1.43)	0.04	1.24 (1.02–1.49)	0.03
Number of antihypertensive medications prescribed	1.40 (1.29–1.52)	<0.0001	1.32 (1.21–1.44)	<0.0001
Interaction term between location of residence and level of health facility			0.82 (0.68–0.99)	0.04

**Any reported difficulty encountered in obtaining antihypertensive medications over the preceding 6 months at the hospital pharmacy

## Discussion

This study to the best of our knowledge is the first in Ghana to robustly evaluate patient-level, health systems-level and access factors influencing blood pressure control among patients receiving care for hypertension. From this multicenter study, we found that 58% of participants had uncontrolled blood pressure. Although >99% of all study participants had valid health insurance cover and were eligible for subsidized essential antihypertensive medicines, one-fifth of study participants reported encountering difficulties accessing these medications at the point of care. Additionally, we found that adherence to hypertensive therapy was not optimal for a significant majority of study participants. Furthermore, knowledge on hypertension and its management among patients was low, despite the average duration of hypertension diagnosis being 8 years. Although urban dwellers were being managed at tertiary hospitals within dedicated hypertension clinics, blood pressure control was poorest in this category compared with rural dwellers receiving care at primary healthcare facilities. Consequently, patient-level factors such as duration of hypertension, compliance with treatment, disease-related factors such as number of antihypertensive medications, and system-level factors such as availability of medications at point of care and the tier of health institution were significantly associated with poor hypertension control.

The rates of blood pressure control in the present study is comparable with that from many other studies in sub-Saharan Africa where control rates rarely exceeded >45% [18–46] although control rates are generally better in the North African countries [47–51]. These trends are generally reflective of the situation in many LMICs outside Africa [52] highlighting poor control of hypertension as major global health issue. In contrast, control rates of hypertension have been shown to be better in European and American cohorts probably due to better awareness and treatment access in these settings [53–55].

Interestingly, although socio-demographic factors such as age, gender, income level and educational status were not significantly associated with hypertension control in the present study, some previous studies have identified associations with these factors albeit inconsistently across studies [56,57]. It is notable that in this random sample of patients attending clinics in Ghana for hypertension care, approximately 75% were females suggesting possible gender differences in health seeking behaviors [58]. This finding is of great concern as it suggests that a significant proportion of males with hypertension in Ghana are either unaware of their diagnosis, are not to receiving treatment or are not compliant with their hypertension treatment, as hypertension is common among African males [59, 60]. Given the recent surge in morbidity and mortality from cardiovascular disease such as stroke in Ghana, [61–63] this gender-related disparity in health seeking behavior among males is a major public health issue requiring urgent attention.

In the Prospective Urban Rural Epidemiology (PURE) study, [55] control of hypertension was found to be better among urban than rural dwellers. We however observed that urban compared with rural dwellers had higher rates of uncontrolled blood pressure probably reflecting the graded increase in the adoption of western lifestyles among urban dwellers. Of note however, in our multivariate model, location of residence was not significantly associated with blood pressure control when we adjusted for the level of health facility where health care was received. These two variables as we have shown are probably inter-related since most participants would seek health care from a health facility closest to their domiciles. It is possible that system level factors that pertain to urban health facilities such as heavy patient load and therefore shorter interaction times between doctors and patients may contribute to these differences. Tertiary hospitals situated in urban settings are more impersonal, and with the constant change of medical staff, limited personal trusting relationships develop between doctors and patients. Furthermore, participants attending tertiary facilities may have different characteristics from those attending primary health facilities. For instance, participants with more difficult to control hypertension may be referred to tertiary centers for treatment.

Lack of health literacy about hypertension was a common theme among study participants, but there were no consistent trends in responses observed between the two groups. Similar studies conducted on knowledge, attitudes and practices in sub-Saharan Africa [64–66] have shown mixed associations between blood pressure control and knowledge base of study participants on hypertension. Since educational attainment was not associated with blood pressure control in the present study, all patients including well-educated ones, should receive targeted education on blood pressure control during clinic visits. It is also imperative that a massive public health initiative aimed at improving awareness and control of hypertension among the general populace be undertaken in the light of the pervasively low health literacy on hypertension in sub-Saharan Africa.

Poor adherence to hypertension treatment captured under three domains namely medication adherence, salt usage and appointment keeping by the Hill Bone questionnaire was as expected a key determinant of blood pressure control. Indeed, for each 5-points higher (higher scores means poor adherence), the risk of poor blood pressure control increased by 21% (p = 0.0006). We found that participants whose blood pressure were not on target reported higher tendencies to miss scheduled clinic appointments, to forget to get prescriptions filled, to run out of medications, to skip medications before coming to clinic and to miss pills when careless. Compositely, medication adherence and appointment keeping scores were significantly better among those with uncontrolled blood pressure but not salt usage which was similar between the two groups. Reasons for non-adherence are multi-factorial and are often contributed to by a mix of patient related, physician related and health system-related factors. This makes adherence to therapy for chronic diseases such as hypertension a major challenge worldwide [67–74]. Hence optimization of adherence to blood pressure treatment would require multimodal approaches which are evidence-based and culturally tailored particularly for individuals in LMICs.

An important finding worth highlighting is the reported difficulties encountered by patients in accessing antihypertensive medications. Unlike the scenario in many LMICs, nearly all study participants in the present study were covered by an insurance scheme which ensured access to essential antihypertensive medications for blood pressure control at the point of care within a hospital pharmacy. However as we have shown, up to 20% of study participants reported encountering difficulties in obtaining medicines, highlighting the frailties of the insurance scheme. Where medications were unavailable, patients would have to obtain medicines from other sources, which required out-of pocket payments for these non-insured medications further increasing the possibility of non-adherence. Hence access and affordability of essential antihypertensive medications for the treatment of hypertension in LMICs may constitute yet another important barrier to blood pressure control.

Although we have attempted to assess various factors that might influence blood pressure control, the study is limited in not assessing health professional level of knowledge on hypertension management. Given our finding that tertiary centers were associated with poor control of hypertension, further investigation is warranted on quality of care provided at the tertiary level of healthcare in Ghana. Other important factors of relevance to blood pressure control such as therapeutic inertia and resistant hypertension were not assessed in the present study. Again, the Hill Bone adherence questionnaire subjectively evaluated the reported adherence of participants and is liable to recall bias. Similarly, history of stroke and heart failure were elicited from participants based on self-reports and are subject to recall bias. We defined current cigarette smoking as smoking a cigarette over the past 12 months but current cigarette smoking is typically assessed as smoking 100 or more cigarettes in lifetime and smoking at least a cigarette over the past 30 days and therefore there is a measurement bias with the corollary of overestimating the frequency of current smokers in our study. Finally the associations observed between the selected variables and blood pressure control in a cross-sectional study design does not connote causal relations.

## Conclusion

In this multi-centre hospital-based study among Ghanaian hypertensive patients, we found more than half (57.4%) of study participants had uncontrolled blood pressure. Health system level factors such as access and affordability of antihypertensive medications at hospital pharmacies and patient-level factors such as adherence to hypertension treatment and knowledge on hypertension should be addressed urgently to prevent the high cardiovascular risk associated with uncontrolled hypertension among Ghanaians.

## Supporting information

S1 Table Minimal dataset used for the current analysis. (XLSX)
